# Prevalence of Sarcopenia in Liver Cirrhosis: A Systematic Review and Meta-Analysis

**DOI:** 10.14309/ctg.0000000000000584

**Published:** 2023-04-03

**Authors:** Simon Mazeaud, Roberta Zupo, Alexis Couret, Francesco Panza, Rodolfo Sardone, Fabio Castellana

**Affiliations:** 1 Clermont Auvergne University, CRNH, AME2P, Clermont-Ferrand, France;; 2 Department of Interdisciplinary Medicine, University “Aldo Moro”, Bari, Italy;; 3 Department of Digestive and Hepatobiliary Medicine, CHU Clermont-Ferrand, Clermont-Ferrand, France;; 4 Department of Translational Biomedicine and Neuroscience (DiBrain), University “Aldo Moro,” Bari, Italy;; 5 Local Healthcare Authority of Taranto, Italy;; 6 Unit of Data Sciences and Technology Innovation for Population Health, National Institute of Gastroenterology IRCCS Saverio De Bellis, Research Hospital, Bari, Italy.

**Keywords:** epidemiology, sarcopenia, liver cirrhosis

## Abstract

Chronic liver disease is often combined with a morbidity burden that strongly affects the functional domain. In liver cirrhosis (LC), qualitative and quantitative muscle wasting, known as sarcopenia, poses an added clinical burden, together with comorbidities and a poor quality of life.

**METHODS:**

We conducted a systematic review and meta-analysis of the prevalence of sarcopenia in LC. The literature was screened through 6 electronic databases from the study's inception to January 2023. No exclusion criteria were applied to language, operative tools for diagnosing sarcopenia, population age, general health status, country, and study setting (cohort or cross-sectional). Two independent researchers applied the inclusion criteria in parallel to evaluate the eligibility of the 44 retrieved articles; only 36 met the eligibility requirements.

**RESULTS:**

The total sample (N = 8,821) was slightly dominated by men (N = 4,941). The cross-sectional design predominated over the longitudinal, and the hospital setting was prevalent. The pooled prevalence of sarcopenia across the selected studies was 33% (95% confidence interval [CI] 0.32–0.34), with high heterogeneity (*I*^2^ = 96%). A further meta-analysis using the Child–Pugh (CP) score to stage LC was conducted on 24 entries, and the results showed that for the LC populations classified with the CP-A, CP-B, and CP-C staging, respectively, the overall mean prevalence was 33% (95% CI 0.31–0.35), 36% (95% CI 0.34–0.39) and 46% (95% CI 0.43–0.50). The risk of bias was moderate. In LC, 1 in 3 patients suffers sarcopenia.

**DISCUSSION:**

Poor management of muscle mass loss plays a role in the prognosis of death and quality of life of patients with LC. Clinicians in the field are recommended, when screening for sarcopenia, to pay close attention by carefully assessing body composition as part of the monitoring scheme.

## Graphical abstract

**Figure:**
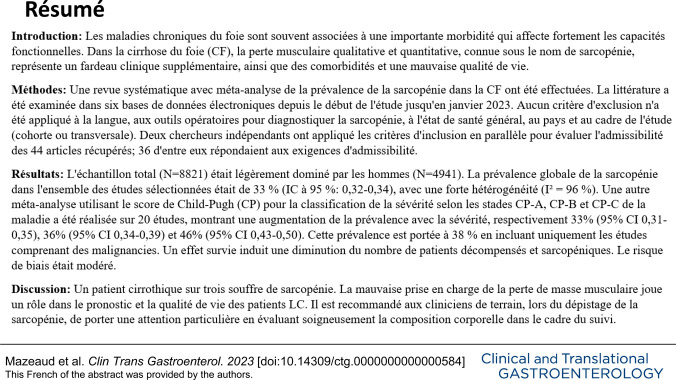

## INTRODUCTION

Chronic diseases currently account for 7 of the top 10 major global causes of death. Likely exacerbated by demographic aging and recent Westernized lifestyle habits, chronic conditions are primarily associated with epigenetic and lifestyle factors, causing a high disease burden, decreased quality of life, and massive healthcare spending. In this context, chronic liver disease (CLD) tops the list of concerns. There is evidence that CLD accounts for 2 million deaths per year worldwide (1), along with a heavy burden of disability, and thus greater disability-adjusted life-years (DALYs) and greater healthcare demands. In 2017, liver cirrhosis (LC) led to more than 1.32 million deaths globally, up from less than 899,000 deaths in 1990 (2,3), accounting for 2.2% of deaths and 1.5% of DALYs worldwide. Epidemiological estimates across several developed countries indicate a prevalence of LC ranging from 4.5% to 9.5% in the general population, approximately 10%–40% of whom undergo a silent, asymptomatic type of LC (4). According to World Health Organization microscopic-level data (5), LC accounts for 1.8% of all deaths in Europe (170,000 per year), with the highest incidence in the southeastern and northeastern regions. However, LC mortality has also increased in the United Kingdom and Ireland in recent years.

Parallel to the increase in life expectancy, the aged population balance is continuously rising (6), along with the burdens of multimorbidity, polypharmacy, and a highly disabling phenotype featuring physical and cognitive decline (6,7). Disease and drug dependency, as well as physiological muscle catabolism and poor taste and smell, play a role in exacerbating a multidimensional aging phenotype featuring loss of physical vigor, muscle mass, and strength, otherwise known as sarcopenia (8). According to the latest 2019 concept, sarcopenia dimensions include low levels of muscle strength, muscle quantity/quality, and of physical performance as an indicator of severity (9). Sarcopenia poses a considerable clinical challenge, especially as the decline approaches multiple irreversible adverse outcomes such as physical disability, dependency, falls, hospitalization, physical frailty, and a reduced quality of life (6,10).

Previous cohort data demonstrated a 2-fold increase in the risk of death and a drop in 5-year survival probability in patients with LC with sarcopenia compared with nonsarcopenic counterparts (11), hence the importance of rapid diagnosis and prevalence data.

In view of the current lack of an epidemiological overview of sarcopenia in CLD, here we conducted a systematic review and meta-analysis on the prevalence of sarcopenia in patients with LC. The aim of this research was to explore the pooled prevalence of sarcopenia in LC settings, with the intent of promoting better clinical management and the implementation of preventive actions, to improve patients' quality of life and reduce healthcare costs.

## METHODS

### Search strategy and selection criteria

**Figure 1.**
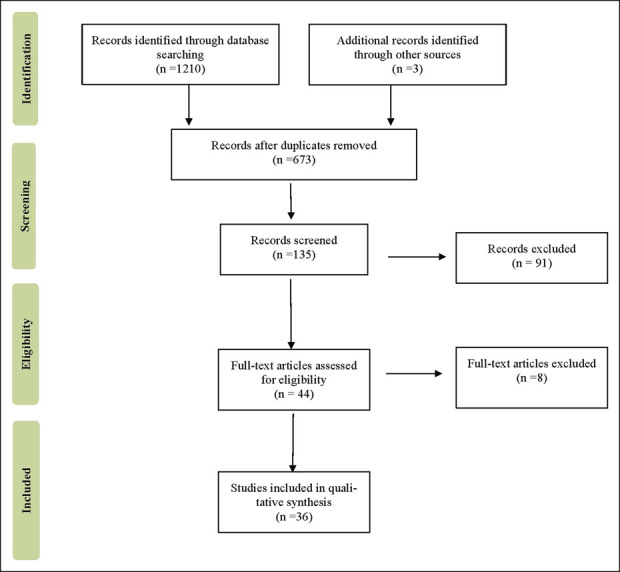
Flow diagram of the literature screening process.

A computerized literature search of MEDLINE and the Cochrane database did not identify any previous systematic reviews on the prevalence of sarcopenia in patients with LC. The present systematic review followed the Preferred Reporting Items for Systematic reviews and Meta-Analyses (PRISMA) guidelines, adhering to the PRISMA 27-item checklist (12) (Figure 1). An *a priori* protocol for the search strategy and inclusion criteria was established and recorded, with no particular changes to the information provided at registration on PROSPERO, a prospective international registry of systematic reviews (CRD42023334706). We performed separate searches in the US National Library of Medicine (PubMed), Medical Literature Analysis and Retrieval System Online (MEDLINE), EMBASE, Scopus, Ovid, and Google Scholar, to find original articles investigating the prevalence of sarcopenia in patients with LC, regardless of the liver disease etiology. The primary objective was to assess the pooled prevalence of sarcopenia in cirrhosis phenotypes of CLD. We also considered the gray literature in the study selection phase, using the huge archive of preprints https://arxiv.org/ and the database http://www.opengrey.eu/ to access abstracts of noteworthy conferences and other unreviewed material. No exclusion criteria were applied to article language, operational constructs used to define the condition of sarcopenia, general health status, country, recruitment context (hospital or nursing home), or study setting (cohort or cross-sectional). As inclusion criteria, we retained only original articles on populations diagnosed with LC, providing some prevalence data on sarcopenia. The presence of malignant tumors was used as a covariate to perform a further meta-analysis based on the presence or absence of malignancy that rapidly exacerbates the pathophysiological paths of sarcopenia.

The research strategy used in PubMed and MEDLINE and adapted to the other 4 electronic sources included the keywords “sarcopenia” and “liver cirrhosis,” combined through the use of Boolean indicators (Table 1). The search strategy used the Boolean indicator NOT to rule out letters, comments, editorials, literature reviews, and meta-analyses. The literature search had no time restrictions, and documents were retrieved until January 31, 2023. No language restrictions were placed. Two researchers (R.Z. and S.M.) searched the articles, reviewed the titles and abstracts of articles retrieved separately and in duplicate, checked the full texts, and selected articles for inclusion in the study. Inter-rater reliability was used to estimate intercoder agreement and then κ statistics to measure accuracy and precision. A κ coefficient of at least 0.9 was obtained in all data extraction steps based on PRISMA concepts and quality assessment steps (13,14).

**Table 1.** Search strategy used in the US National Library of Medicine (PubMed) and Medical Literature Analysis and Retrieval System Online (MEDLINE) and adapted to the other sources, according to selected descriptors

Research question	How prevalent is sarcopenia in liver cirrhosis?
Search concepts	Liver cirrhosis, Sarcopenia
Sources	PubMed, MEDLINE, EMBASE, Scopus, Ovid, and Google Scholar
Limitations	“Human”
Grey literature	https://arxiv.org/. Furthermore, https://www.base-search.net/ was used to avoid publication bias in terms of contradictory and negative results' reports, especially in a gray research question such as the one we selected
Search date	Inception (2001) to January 2023

N	Searches	Results
#1	Sarcopenia	15,052
#2	Liver cirrhosis OR Liver disease	804,467
#3	Review OR Systematic Review OR Meta-analysis OR Comment OR Editorial	5,243,606
#4	#1 AND #2 NOT #3	667

### Data elaboration and analysis

Two researchers (S.M., A.C., and R.Z.) extracted the following information separately and in duplicate in a piloted form: author(s), year of publication, region (country), study design (longitudinal or cross-sectional), study setting (hospital or care center), type of CLD, and clinical tool (bioimpedance [BIA], computed tomography [CT] scan, dual-energy x-ray absorptiometry [DXA], magnetic resonance imaging [MRI], dynamometry, and others) and operative construct adopted to assess sarcopenia across each selected study. Researchers tabulated data by sarcopenia prevalence in patients with LC to retrieve information on (i) sample size (N); (ii) age (expressed as mean ± SD, or interquartile range, or just as a range); (iii) sarcopenia events (N, %); (iv) male and female representativeness (expressed as N and %) in the whole sample and in the sarcopenia subset; (v) operational construct of sarcopenia used and cutoff values applied for each dimension; and (vi) tools for body composition assessment. All references selected for retrieval from the databases were managed with the MS Excel data collection software platform by an experienced biostatistician (F.C.). Finally, the data extracted from the selected studies and stored in the database were structured as evidence tables.

The tool developed by Hoy et al (14) was adopted in the present research to assess whether the 35 selected research studies were conducted according to the highest possible standards (methodological quality), and the degree of credibility of the results (risk of bias). Each study was assigned a score of 1 (yes) or 0 (no) for each of the 10 criteria. Based on the total score, studies were classified as at low (>8), moderate (6–8), or high (≤5) risk of bias. Disagreements between the 2 researchers on the methodological quality of the included studies were discussed until an agreement with a third researcher (R.S.) was reached.

**Figure 2.**
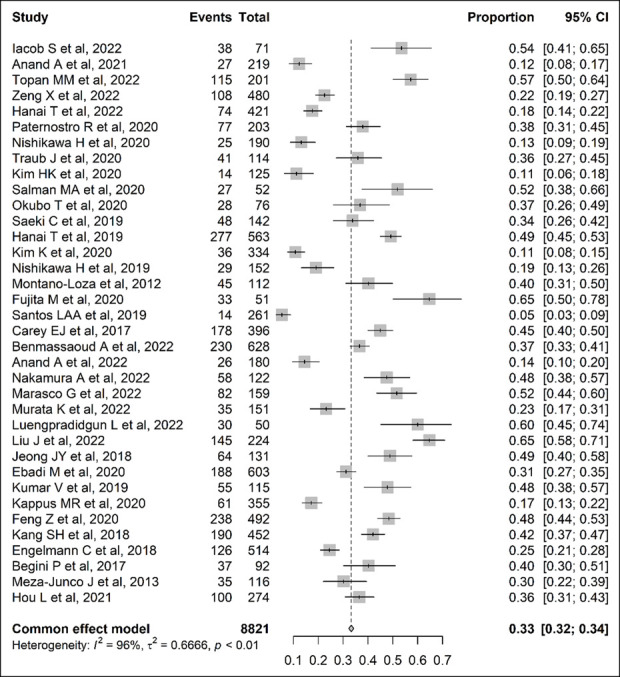
Forest plot of studies estimating the proportion of sarcopenic individuals in patients with cirrhosis. CI, confidence interval.

**Figure 4.**
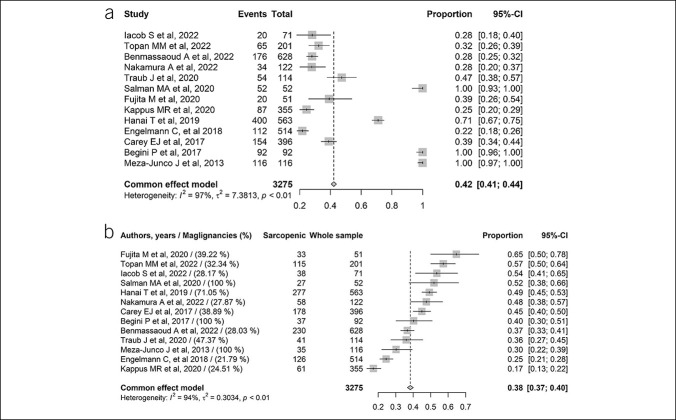
(**a**) Forest plot for malignancies prevalence in selected studies. (**b**) Forest plot for sarcopenia prevalence regarding to malignancy prevalence. CI, confidence interval.

Meta-analyses were performed using the “Metaprop” function in the R package “meta” (version 5.2.0; R Foundation for Statistical Computing, Vienna, Austria). To estimate the overall pooled prevalence of sarcopenia in patients with LC, DerSimonian–Laird random-effect meta-analyses were conducted using the inverse variance method (Figure 2). The logit transformation was applied to stabilize the variance and normalize its distribution. The Clopper–Pearson method was used to calculate point estimates and 95% confidence intervals (CIs) for the prevalence rate of sarcopenia in each study. Statistical heterogeneity was measured by the *I*^2^ statistic (15), and values less than 25%, between 25% and 75%, and more than 75% were taken to show low, medium, and high heterogeneity, respectively (15). Subgroup analysis was performed on the basis of potential sources of heterogeneity, such as LC staging, which has been acknowledged as a powerful contributor to the development of sarcopenia. So, we used the Child–Pugh (CP) score to divide the cirrhotic population into 3 groups based on the severity and complexity of the LC status, that is, CP-A, CP-B, and CP-C (Figure 3a). The upward profile of sarcopenia prevalence in relation to the severity of LC by CP was further described for each study using a dodge plot (Figure 3b). Finally, a subgroup of 13 studies that provided prevalence data of sarcopenia in subjects with LC complicated by malignancies allowed us to conduct an additional meta-analysis to appreciate the variation of sarcopenia prevalence in those individuals to compare with the malignancy-free counterparts. Figure 4a shows the proportion of malignancies in LC, whereas Figure 4b shows the proportion of malignancies in LC by subgroups of sarcopenia (presence/absence). All data analyses were performed by a senior biostatistician (F.C.) using R, version 2021.09.1.

Figure 3.(**a**) Forest plot of studies estimating the proportion of Child–Pugh (A, B, C) in liver cirrhosis individuals. (**b**) Forest plot of studies estimating the prevalence of sarcopenia in liver cirrhosis according to Child–Pugh (A, B, C) groups. (**c**) Dodge plot for prevalence of sarcopenia in liver cirrhosis according to Child–Pugh (A, B, C) groups across studies. CI, confidence interval.
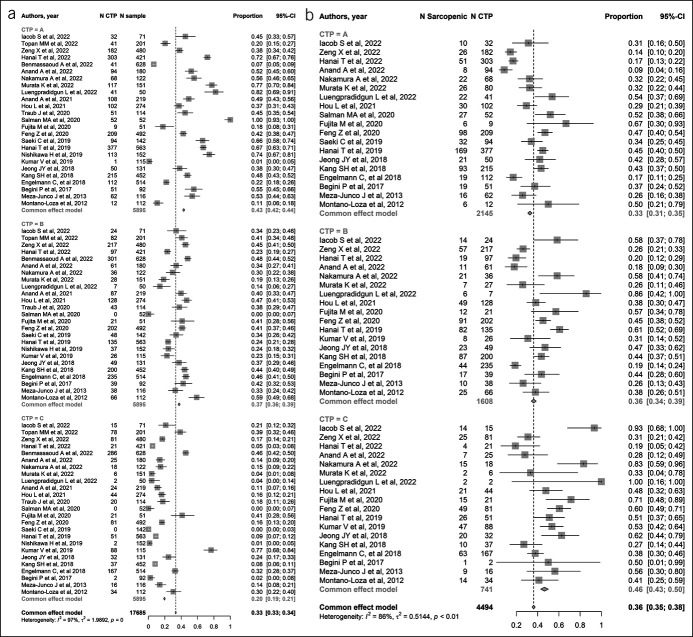

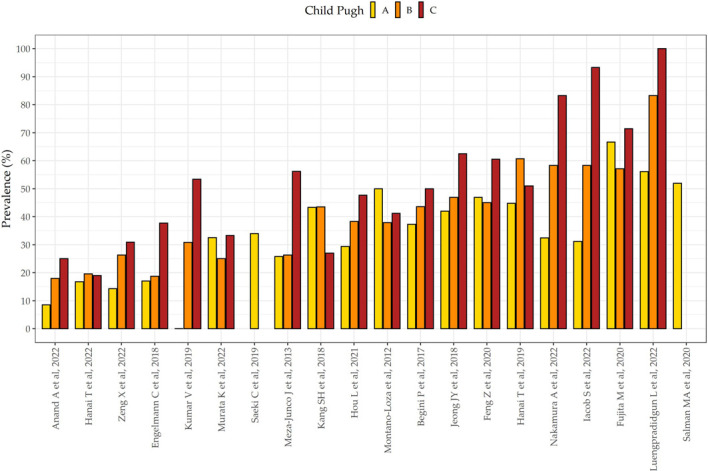


## RESULTS

The first systematic literature search yielded 667 entries (Table 1). After excluding duplicates, 77 were classified as potentially relevant and selected for the title and abstract analysis. Then, 37 were excluded because they did not meet the characteristics of the approach or the objective of this research. After reviewing the full text of the remaining 40 articles, only 36 met the inclusion criteria and were included in the meta-analysis (16–51). The PRISMA flow chart illustrating the number of studies at each stage of the review is shown in Figure 1.

The final research focus included 36 articles reporting the prevalence of sarcopenia in LC populations. Table 2 shows details of the design (cohort or cross-sectional), setting (hospital or nursing home), sample size (N), type of sarcopenia construct used to assess the prevalence (including cutoffs for each dimension), and body composition assessment tool (bioimpedance, DXA, CT scan, MRI, and dynamometry). The cross-sectional design (80.5%, N = 29 of 36) predominated over the longitudinal design (19.5%, N = 7 of 36). In all cases, the study setting was the hospital, except for 1 study conducted at a care center. The geographical distribution of the studies favored Asia (61%, N = 22), followed by Europe (22%, N = 8), America (14%, N = 5), and Africa (3%, N = 1). In accordance with the inclusion criteria, all subjects had LC.

**Table 2.** Descriptive of selected studies investigating the prevalence of sarcopenia in liver cirrhosis (N = 36)

Study	Region (country)	Age	Study design	Study setting	Diagnosis of sarcopenia	Method of body composition assessment	Score (risk of bias)
Iacob et al, 2022 (16)	Romania (Europe)	54.5 ± 12.6	Cross-sectional	Hospital	EWGSOP2 criteria combining low HGS (<27 kg for men and HGS <16 kg for women) with low L3-SMI (<50 cm^2^/m^2^ for men and <39 cm^2^/m^2^ for women)	CT scan and dynamometry	7 (moderate)
Anand et al, 2022 (17)	India (Asia)	18–60 (mean 42.6)	Cohort	Care centre	EWGSOP2 criteria combining low HGS (<27 kg for men and HGS < 16 kg for women) with low L3-SMI (<50 cm^2^/m^2^ for men and <39 cm^2^/m^2^ for women)	CT scan and dynamometry	7 (moderate)
Topan et al, 2022 (18)	Romania (Europe)	61.65 ± 9.49	Cohort	Hospital	EWGSOP2 criteria combining low HGS (<27 kg for men and HGS <16 kg for women) with low L3-SMI (<50 cm^2^/m^2^ in men and <39 cm^2^/m^2^ in women)	CT scan and dynamometry	7 (moderate)
Zeng et al, 2022 (19)	China (Asia)	18 to 80	Cross-sectional	Hospital	Low L3-SMI (<44.77 cm^2^/m^2^ for men and <32.50 cm^2^/m^2^ for women)	CT scan	7 (moderate)
Hanai et al, 2021 (20)	Japan (Asia)	71 (64–78)	Cross-sectional	Hospital	JSH criteria combining low HGS (<26 kg for men and <18 kg for women) with low SMI (<7.0 kg/m^2^ for men and <5.7 kg/m^2^ for women)	CT scan	7 (moderate)
Paternostro et al, 2020 (21)	Austria (Europe)	18+	Cohort	Hospital	Low TPMT‐L3 (<12 mm/m for men and <8 mm/m for women)	CT/MRI	7 (moderate)
Nishikawa et al, 2020 (22)	Japan (Asia)	50–72	Cross-sectional	Hospital	JSH criteria combining low HGS (<26 kg for men and <18 kg for women) with low SMI (<7.0 kg/m^2^ for men and <5.7 kg/m^2^ for women)	BIA and dynamometry	7 (moderate)
Traub et al, 2020 (23)	Austria (Europe)	65 (61.87–65.97)	Cross-sectional	Hospital	EWGSOP criteria combining low HGS (<30 kg for men and <20 kg for women) with low L3-SMI (<50 cm^2^/m^2^ in men and <39 cm^2^/m^2^ in women)	CT scan and dynamometry	7 (moderate)
Kim et al, 2020 (24)	Korea (Asia)	55.9 ± 10.9	Cross-sectional	Hospital	Low SI (total ASM [kg]/BMI [kg/m^2^] <0.789 in men and <0.521 in women)	BIA	7 (moderate)
Salman et al, 2020 (25)	Egypt (Africa)	53.9 ± 5.0	Cohort	Hospital	Low L3-SMI (≤53 cm^2^/m^2^ for men with a BMI ≥25 and ≤43 cm^2^/m^2^ for men with BMI <25 and ≤41 cm^2^/m^2^ for women, irrespective of BMI)	CT scan	7 (moderate)
Okubo et al, 2020 (26)	Japan (Asia)	67 (24–8)	Cross-sectional	Hospital	JSH criteria combining low HGS (<26 kg for men and <18 kg for women) with low SMI (<7.0 kg/m^2^ for men and <5.7 kg/m^2^ for women)	BIA and dynamometry	7 (moderate)
Saeki et al, 2019 (27)	Japan (Asia)	70.5 (58.8–76.0)	Cross-sectional	Hospital	JSH criteria combining low HGS (<26 kg for men and <18 kg for women) and/or low gait speed (≤0.8 m/sec both for men and women) and low SMI (<7.0 kg/m^2^ for men and <5.7 kg/m^2^ for women)	BIA, dynamometry and gait speed	7 (moderate)
Hanai et al, 2019 (28)	Japan (Asia)	71 ± 11	Cohort	Hospital	JSH criteria combining low HGS (<26 kg for men and <18 kg for women) with low L3-SMI (≤42 cm^2^/m^2^ for men and ≤38 cm^2^/m^2^ for women)	CT scan and dynamometry	7 (moderate)
Kim et al, 2020 (29)	Korea (Asia)	54.4 ± 12.7	Cross-sectional	Hospital	Low SI (total appendicular skeletal muscle mass [kg]/BMI [kg/m^2^] <0.789 for men and <0.521 for women)	BIA	7 (moderate)
Nishikawa et al, 2019 (30)	Japan (Asia)	61.5 ± 12.7	Cross-sectional	Hospital	JSH criteria combining low HGS (<26 kg for men and <18 kg for women) and low SMI (<7.0 kg/m^2^ for men and <5.7 kg/m^2^ for women)	BIA and dynamometry	7 (moderate)
Montano-Loza et al, 2012 (31)	Canada (America)	54 ± 1	Cohort	Hospital	Low L3-SMI (≤52.4 cm^2^/m^2^ for men and ≤38.5 cm^2^/m^2^ for women)	CT scan	7 (moderate)
Fujita et al, 2020 (32)	Japan (Asia)	Not reported	Cohort	Hospital	Low PMI-L3 (<6.0 for men and <3.4 cm^2^/m^2^ for women)	Computed tomography (CT) scan	7 (moderate)
Santos et al, 2019 (33)	China (Asia)	57 (51.75–63.00)	Cohort	Hospital	EWGSOP criteria combining low HGS (<30 kg for men and <20 kg for women) with low SMI (<7.26 kg/m^2^ for men and <5.45 kg/m^2^ for women)	DXA and dynamometry	7 (moderate)
Carey et al, 2017 (34)	USA (America)	58 (51–62)	Cohort	Hospital	Low L3-SMI (<50 cm^2^/m^2^ for men and <39 cm^2^/m^2^ for women)	CT scan	7 (moderate)
Benmassaoud et al, 2022 (35)	United Kingdom (Europe)	54.5 ± 14	Cohort	Hospital	Low L3-SMI (<50 cm^2^/m^2^ for men and <39 cm^2^/m^2^ for women)	CT scan	7 (moderate)
Anand et al, 2021 (36)	India (Asia)	18–60	Cross-sectional	Hospital	Low L3-SMI (<36.5 cm^2^/m^2^ for men and <30.2 cm^2^/m^2^ for women)	CT scan	7 (moderate)
Nakamura et al, 2022 (37)	Japan (Asia)	62 ± 14	Cross-sectional	Hospital	Low PSMI (<12.62 cm^2^/m^2^ for men and <9.77 cm^2^/m^2^ for women)	MRI	7 (moderate)
Marasco et al, 2022 (38)	Italy (Europe)	68 (median)	Cohort	Hospital	Low L3-SMI (<50 cm^2^/m^2^ for men and <39 cm^2^/m^2^ for women)	CT scan	7 (moderate)
Murata et al, 2022 (39)	Japan (Asia)	70 ± 10	Cross-sectional	Hospital	JSH criteria combining low HGS (<26 kg for men and <18 kg for women) with L3-SMI (≤42 cm^2^/m^2^ for men and ≤38 cm^2^/m^2^ for women)	CT scan and dynamometry	7 (moderate)
Luengpradidgun et al, 2022 (40)	Thailand (Asia)	63 (54.5–64.5)	Cross-sectional	Hospital	JSH criteria combining low HGS (<26 kg for men and <18 kg for women) with L3-SMI (≤42 cm^2^/m^2^ for men and ≤38 cm^2^/m^2^ for women)	CT scan and dynamometry	7 (moderate)
Liu et al, 2022 (41)	China (Asia)	54.3 ± 11.6	Cohort	Hospital	Low L3-SMI (<50 cm^2^/m^2^ for men and <39 cm^2^/m^2^ for women)	CT scan	7 (moderate)
Jeong et al, 2018 (42)	Korea (Asia)	53.7 ± 9.6	Cohort	Hospital	Low L3-SMI (≤52.4 cm^2^/m^2^ for men and ≤38.5 cm^2^/m^2^ for women)	CT scan	6 (moderate)
Ebadi et al, 2020 (43)	Canada (America)	18–40	Cross-sectional	Hospital	Low L3-SMI (<42 cm^2^/m^2^ for men and <30 cm^2^/m^2^ for women)	CT scan	7 (moderate)
Kumar et al, 2019 (44)	India (Asia)	45.75 ± 10.6	Cross-sectional	Hospital	Low L3-SMI (≤52.4 cm^2^/m^2^ for men and ≤38.5 cm^2^/m^2^ for women)	CT scan	7 (moderate)
Kappus et al, 2020 (45)	USA (America)	53.7 ± 12.0	Cohort	Hospital	Low L3-SMI (<50 cm^2^/m^2^ for men and <39 cm^2^/m^2^ for women)	CT scan	7 (moderate)
Feng et al, 2020 (46)	China (Asia)	51 (45–61)	Cohort	Hospital	Low L3-SMI (<50 cm^2^/m^2^ for men and <39 cm^2^/m^2^ for women, according to EWGSOP2 criteria) or low SMD (myosteatosis) according to SMD <34.1 HU for men and <27.2 HU for women	CT scan	7 (moderate)
Kang et al, 2018 (47)	Korea (Asia)	51.8 ± 8.8	Cohort	Hospital	Low L3-SMI (≤52.4 cm^2^/m^2^ for men and ≤38.5 cm^2^/m^2^ for women)	CT scan	6 (moderate)
Engelmann et al 2018 (48)	Germany (Europe)	53.7 (mean)	Cohort	Hospital	Low L3-SMI (<41.90 cm^2^/m^2^ for men and <35.30 cm^2^/m^2^ for women)	CT scan	7 (moderate)
Begini et al, 2017 (49)	Italy (Europe)	71.6 (30.7–86.4)	Cohort	Care centre	SMI ≤41 cm^2^/m^2^ for women and ≤53 cm^2^/m^2^ for men with BMI ≥25, and ≤43 cm^2^/m^2^ for men and women with BMI <25, respectively	CT scan	7 (moderate)
Meza-Junco et al, 2013 (50)	Canada (America)	58 ± 6	Cohort	Hospital	Low L3-SMI: ≤41 cm^2^/m^2^ for women and ≤53 cm^2^/m^2^ for men with body BMI ≥25 and ≤43 cm^2^/m^2^ in patients with BMI <25	CT scan	7 (moderate)
Hou et al, 2021 (51)	China (Asia)	62.2 ± 12.9	Cohort	Hospital	Low L3-SMI (<46.9 cm^2^/m^2^ for men and <32.5 cm^2^/m^2^ for women)	CT scan	7 (moderate)

BIA, bioelectrical impedance analysis; BMI, body mass index; CT, computed tomography; DXA, dual-energy x-ray absorptiometry; EWGSOP, European Working Group on Sarcopenia in Older People; HGS, hand grip strength; JSH, Japan Society of Hepatology; L3, third lumbar vertebra; MRI, magnetic resonance imaging; PMI, psoas muscle index; PSMI, paraspinal muscle index; SI, sarcopenia index; SMD, skeletal muscle radiodensity; SMI, skeletal muscle index; TPMT, transversal psoas muscle thickness.

As regards the concepts of sarcopenia used, most prevalence entries (16–20,22–28,30,31,33–51) were based on deficiency values of the third lumbar vertebra skeletal muscle index (L3-SMI) assessed by CT scan. Eight entries (20,22,26,27,30,39,40) followed the Japan Society of Hepatology guidelines for sarcopenia in liver disease (52), with threshold values for muscle mass depending on the type of clinical device, BIA, DXA, or CT scan. Then, the criteria of the European Working Group on Sarcopenia in Older People, updated to 2019 (9), were applied by 4 studies (16–18,46), whereas 2 (23,33) relied on the first European Working Group on Sarcopenia in Older People consensus (53). Here, cutoffs for muscle mass and strength differed according to the type of population, whether Asian (17) or Western (23). Two entries by Kim et al (24,29) applied the sarcopenia index, that is, the ratio of total appendicular skeletal muscle mass (kg) to body mass index (kg/m^2^), using consistent cutoffs of <0.789 for men and <0.521 for women. Two sarcopenia entries Resulted from muscle deficits estimated by the psoas muscle index of L3 (32,35). Finally, a single prevalence entry was derived from the construct used by (37), namely, a paraspinal muscle index deficiency, diagnosed on values <12.62 cm^2^/m^2^ for men and <9.77 cm^2^/m^2^ for women.

As an assessment tool for body composition, most studies used CT scans to quantify muscle mass (Table 2). Only 6 sarcopenia entries were derived from BIA (22,24,26,27,29,30,54) and just 1 from DXA (33) and 2 for MRI (21,37). For handgrip strength estimation, dynamometry was chosen in 12 studies to estimate the prevalence of sarcopenia, combined with appendicular muscle mass. Saeki et al (27) also used walking speed as a substitute estimate for muscle loss/exhaustion.

Table 3 shows data on sample size and sarcopenia prevalence subdivided by gender if provided. A total sample of 8,821 subjects with LC was analyzed, including a slight majority of men (N = 4,941). Of note, 4 studies did not provide quantitative data on the gender ratio and thus were not accounted for in the total female and male subsets' count. Sarcopenia prevalence rates (%) were recorded in Table 3, and the gender ratio and CP (A, B, and C) staging proportion was also provided. The prevalence of sarcopenia across selected studies, accounting for 8,821 patients with LC, showed a pooled average prevalence of 33% (95% CI 0.32–0.34) (Figure 2), but high heterogeneity, *I*^2^ = 96%. Sex analysis showed a larger sarcopenia prevalence in males than women, respectively 49.2% and 23.6% (data not shown).

**Table 3.** Overview of sample size, gender ratio, the prevalence of sarcopenia, and cirrhosis severity by Child–Pugh scoring across selected studies

Study	Sarcopenia events (N)	Sample size (N)	Male (N)	Female (N)	Prevalence of sarcopenia (%)	Male sarcopenia events (N)	Female sarcopenia events (N)	Child–Pugh A (N)	Child–Pugh B (N)	Child–Pugh C (N)
Iacob et al, 2022 (16)	38	71	48	23	53.5	Not reported	Not reported	32	24	15
Anand et al, 2022 (17)	27	219	168	51	12.3	24	3	Not reported	Not reported	Not reported
Topan et al, 2022 (18)	115	201	127	294	57.2	76	39	41	82	78
Zeng et al, 2022 (19)	108	480	286	184	22.5	86	22	182	217	81
Hanai et al, 2021 (20)	74	421	Not reported	Not reported	17.6	Not reported	Not reported	303	97	21
Paternostro et al, 2020 (21)	77	203	138	65	37.9	62	15	Not reported	Not reported	Not reported
Nishikawa et al, 2020 (22)	25	190	103	87	13.1	12	13	Not reported	Not reported	Not reported
Traub et al, 2020 (23)	41	114	86	28	36.0	36	5	51	43	20
Kim et al, 2020 (24)	14	125	Not reported	Not reported	11.2	Not reported	Not reported	Not reported	Not reported	Not reported
Salman et al, 2020 (25)	27	52	38	14	51.9	18	9	52	0	0
Okubo et al, 2020 (26)	28	76	41	35	36.8	19	9	Not reported	Not reported	Not reported
Saeki et al, 2019 (27)	48	142	90	52	33.8	26	22	94	48	0
Hanai et al, 2019 (28)	277	563	375	188	49.2	221	56	377	135	51
Kim et al, 2020 (29)	36	334	Not reported	Not reported	10.8	Not reported	Not reported	Not reported	Not reported	Not reported
Nishikawa et al, 2019 (30)	29	152	Not reported	Not reported	19.1	Not reported	Not reported	113	37	2
Montano-Loza et al, 2012 (31)	45	112	78	34	40.2	39	6	12	66	34
Fujita et al, 2020 (32)	33	51	26	25	64.7	21	12	9	21	21
Santos et al, 2019 (33)	14	261	161	100	5.36	Not reported	Not reported	Not reported	Not reported	Not reported
Carey et al, 2017 (34)	178	396	277	119	45	139	39	Not reported	Not reported	Not reported
Benmassaoud et al, 2022 (35)	230	628	429	199	36.60	177	53	42	300	286
Anand et al, 2021 (36)	26	180	143	37	14.4	21	5	94	61	25
Nakamura et al, 2022 (37)	58	122	75	47	47.5	Not reported	Not reported	68	36	18
Marasco et al, 2022 (38)	82	159	128	31	51.6	68	14			
Murata et al, 2022 (39)	40	151	95	56	26.5	Not reported	Not reported	117	28	6
Luengpradidgun et al, 2022 (40)	30	50	28	22	60.0	11	19	41	6	3
Liu et al, 2022 (41)	145	224	159	65	64.7	113	32	Not reported	Not reported	Not reported
Jeong et al, 2018 (42)	64	131	94	37	48.9	55	9	50	49	32
Ebadi et al, 2020 (43)	188	603	408	195	31.2	153	35	Not reported	Not reported	Not reported
Kumar et al, 2019 (44)	55	115	104	11	47.8	51	4	1	26	88
Kappus et al, 2020 (45)	61	355	232	123	17.2	57	4	Not reported	Not reported	Not reported
Feng et al, 2020 (46)	238	492	365	127	48.4	183	55	209	202	81
Kang et al, 2018 (47)	190	452	379	73	42	178	12	215	200	37
Engelmann et al 2018 (48)	126	514	363	151	24.50	—	—	112	235	167
Begini et al, 2017 (49)	37	92	27	65	40.20	20	17	51	39	2
Meza-Junco et al, 2013 (50)	35	116	98	18	30.20	30	5	62	38	16
Hou et al, 2021 (51)	100	274	144	130	36.50	70	30	102	128	44

Figure 3a showed the prevalence of CP-A, CP-B, and CP-C events in the LC population. The findings reinforced the internal validity of our meta-analysis and showed a downtrend of subjects moving from the less- to the more-complicated LC staging (survival effect) as follows: 43% (95%CI 0.42–0.45), 37% (95%CI 0.36–0.39), and 20% (95%CI 0.19–0.21) of LC subjects fell into the CP-A, CP-B, and CP-C groups, respectively. Then, based on the CP group-scoring meta-analysis, the prevalence of sarcopenia was distributed as follows: 33% (95%CI 0.31–0.35), 36% (95%CI 0.34–0.39), and 46% (95%CI 0.43–0.50) in CP-A, CP-B, and CP-C groups, respectively (Figure 3b), thus showing a slight uptrend in accordance with the severity of the cirrhotic disease.

Figure 4a, b showed the findings of a further meta-analysis on a subgroup of LC subjects whose malignancies and sarcopenia prevalence data were provided contextually by authors. Malignancy prevalence was 42% (95%CI 0.41–44, I^2^=97%) and the associated prevalence of sarcopenia was increased to 38% (95%CI 0.37–40, I^2^=94%). However, the amount of data is still sparse (only 13 studies, N=3275) and thus leaves room for further research.

About other results (data not shown), the whole cohort (25 studies, n=6013) MELD score estimation was 12.4. It was increased to 12.8 for the sarcopenic cohort (20 studies, n=1904) and decreased in the non-sarcopenic cohort (20 studies, n=3013) to 11.8. The most common etiology of LC (29 studies, n=7044) was viral (42.1%) followed by alcohol (28.9%), NAFLD (7.8%) then 21.2% for other etiologies. Regarding complications, ascites, upper gastro-instestinal, esophageal varices, hepatic encephalopathy and bacterial peritonitis were 55.4% (20 studies), 43.5% (9 studies), 69.7% (6 studies), 35.3% (21 studies), 15.3% (6 studies) prevalent, respectively.

## DISCUSSION

The present research was undertaken to provide a revised estimate of the prevalence of sarcopenia in LC. Given the significantly increased burden of disease-related complications, worse quality of life (and increased DALYs), healthcare costs, and shortened survival in patients with LC, the appraisal of sarcopenia calls for a raised awareness of the importance of screening and clinical management.

Meta-analysis in this study of 36 entries involving 8,821 patients with LC, resulted in an overall estimated prevalence of sarcopenia of 33% (95% CI 0.32–0.34), with a high *I*^2^ = 96% heterogeneity, and a moderate risk of bias across selected reports. With a lower population (N=6403) patients, Tantai et al (11) found a higher overall prevalence (37.5%), justifiable by their slight higher proportion of compensated LC (37.5% CP-A; 43.3% CP-B; 19.1% CP-C; N=3048) compared to us (43% CP-A; 37% CP-B; 20% CP-C; N=5895). Thus, our results seem to be closer to a “real world” prevalence whereas theirs seem more “clinical world”. Based on the CP severity of LC, our findings indicated a prevalence of sarcopenia distributed as follows: 33% (95%CI 0.31–0.35), 36% (95%CI 0.34–0.39), and 46% (95%CI 0.43–0.50) in CP-A, CP-B, and CP-C groups, respectively, thus showing a slight uptrend in accordance with the severity of the cirrhotic disease. This result was in accordance with Tantai et al (11): 28.3%, 37.9% and 46.7%. Regarding differences between men and women, our results indicated 23.6% vs 49.2% while Tantai et al (11) found 28.7% vs 41.9% and Kim et al (55) found 36% vs 61.6%. To this observation, a multicentrique study by Michitaka et al (56) showed that alcoholic etiology in LC men is almost fivefold more prevalent than in women, which is more associated with sarcopenia. Furthermore, our overall population was slightly more masculine, which is very common in LC. In our subgroup analysis, malignancy prevalence was 42% (95%CI 0.41–44, I^2^=97%) representing 1382 subjects, and the associated prevalence of sarcopenia was increased to 38% (95%CI 0.37–40, I^2^=94%). Despite the amount of data (only 13 studies, N=3275) and a non-exclusively HCC cohort, our results seem to be in accordance with the meta-analysis of Guo et al (57) (41.7%).

From a pathophysiological point of view, sarcopenia is recognized to involve multidomain pathways, ultimately leading to a failure of the balance between protein synthesis and breakdown. Through metabolic and biochemical abnormalities, CLD is known to disrupt whole-body protein homeostasis and directly reduce muscle retention. Multiple metabolic pathways, including those listed as follows, are believed to be incriminated (58). Hyperammonemia, a common feature of LC, has the potential to increase myostatin activity and impair mitochondrial function (59,60). Proinflammatory cytokines, such as tumor necrosis factor-α and nuclear factor-κB signaling, glucocorticoids, and impaired insulin-like growth factor-1 signaling paths are well known to boost proteasome activity and autophagy (61). In addition, the increased hepatic gluconeogenesis shared by LC sufferers, and likely due to limited hepatic glycogen content and insulin resistance, may decrease the availability of branched-chain amino acids and glucose to myocytes. Moreover, recent clinical trials have found a lack of testosterone in subjects with LC, pointing to increased muscle cell apoptosis and myostatin activity (62).

Muscle atrophy may also be induced by chronic catabolic conditions such as cancer cachexia, increased energy expenditure, decreased food intake because of loss of appetite, early satiety, side effects of treatment, or changes in gastrointestinal motility, as well as changes in hormone levels such as insulin and catecholamines (8,63). This latter facet is the most relevant in justifying the prevalence gap in sarcopenia rates found between the malignancy subset of LC and their counterparts. Furthermore, atrophy of type II fast-twitch glycolytic fibers, which underlies the development of sarcopenia, may occur in patients with LC (58). Other lifestyle factors associated with LC may indirectly impair the nitrogen balance, especially a poor diet (low protein and calorie intake), low activity levels, and a sedentary lifestyle (64).

Preservation of muscle mass, avoiding rapid loss of mass and transition to sarcopenia, seems to be crucial for the vital prognosis of patients with LC, and indeed, loss of muscle mass is one of the best predictors of death (31). Among patients with LC, a low total SMI is significantly associated with a worse prognosis, whereas higher SMI scores for the arms and legs are associated with a better prognosis (65). Against this background, it should be a priority in the clinical management of LC to monitor body composition and screen for sarcopenia to improve survival and quality of life.

So far, successful intervention trials performed in these settings have relied primarily on nutritional therapies, exercise programs, and testosterone therapy. In terms of muscle health response, these therapies induced a significant improvement in muscle mass/strength and quality of life in each intervention group (66–68). For practical purposes, Hayashi et al (64) suggested that walking 5,000 or more steps per day and maintaining a total energy intake of 30-kcal/ideal body weight can serve as reference lifestyle guidelines for compensated patients with LC. Tandon et al (69) suggest a personalized, moderately low-calorie diet (∼500–800 kcal/d) in obesity settings. They also suggest a protein intake of 1.2- to 1.5-g/kg body weight per day up to 2.0-g/kg body weight per day, depending on the severity of sarcopenia, emphasizing branched-chain amino acid intake. Regarding exercise, Duarte-Rojo et al (68) suggest that 30- to 60-minute sessions combining both aerobic and resistance training for a total of ≥150 min/wk is a reasonable recommendation. A very recent report from Aamann et al (70) indicated people with LC who train die less and goes less to hospitals.

We acknowledge some limitations of this meta-analysis that may directly impact the prevalence results.

Regarding instruments and diagnosis, it is noteworthy that Sinclair et al (71) found significant differences in the prevalence of sarcopenia when applying CT scan (70.3%) or DXA (38.7%). Furthermore, Buchard et al (72) indicated MRI and CT scan as the only reliable devices for cirrhotic patients with ascites (CP-B and CP-C) and potentially DXA for compensated patients (CP-A). Depending on the definition used, Da Silva et al (73) found more or less incident sarcopenia. However, this overview lacks consistency in the clinical device used for body composition assessment (MRI, DXA, dynamometer, BIA, and CT) and the construct used for diagnosing sarcopenia across studies. Also, we lacked to describe the tools and criteria used for assessing LC.

On the other hand, drugs, operations, or other treatments were rarely reported. In some of our selected studies, patients with LC were a subset of a larger population, which could create a bias. Another significant limitation might be related to the etiology of LC. Finally, Bhanji et al (74) reported that patients with nonalcoholic steatohepatitis, the most advanced stage of nonalcoholic fatty liver disease, have a significantly lower prevalence of sarcopenia (22%) than patients with alcoholic liver disease (47%). In fact, these patients have a higher body mass index and obesity, fat deposits, and utilization that could have a muscle reserve effect and induce an anticatabolic environment (75), and may require more muscle work for the same exercise (76), whereas alcohol consumption leads to anabolic muscle resistance (77). However, patients with nonalcoholic steatohepatitis have a 6-fold higher risk of sarcopenic obesity than alcoholics (78). Therefore, exploring the moderating effect of the nonalcoholic fatty liver disease etiology vs sarcopenia among patients with LC with prevalence data could offer a further study focus.

Avoiding comorbidities in patients with LC may lower the risk of death and disease-related disability, especially muscle wasting, physical decline, and sarcopenia. This research stresses the utility of considering sarcopenia as a critical comorbidity in liver disease because 1 in 3 patients with LC is affected. Furthermore, the severity of LC impacts negatively, indeed, the prevalence increases in CP A-B-C respectively, (33%–36%–46%), likely due to the drug therapy and disease-induced muscle-catabolism environment. There is an urgent necessity to assess sarcopenia systematically in LC patients and to recommend lifestyle interventions to reduce risk.

## CONFLICTS OF INTEREST

**Guarantors of the article:** Fabio Castellana, MS, and Roberta Zupo, MS.

**Specific author contributions:** S.M., R.S., and R.Z.: designed the study, performed searches, extracted data, assessed data quality, performed statistical analyses, and wrote the manuscript. S.M., F.C., A.C., and R.Z.: performed searches, extracted data, assessed data quality, and reviewed the manuscript. F.C.: assisted with statistical analyses, performed the analysis, and reviewed the manuscript. R.S.: reviewed the manuscript. R.S. provided input into study design and analysis and reviewed the manuscript.

**Financial support:** None to report.

**Potential competing interests:** None to report.
